# Long-Term Effects on the Lipidome of Acute Coronary Syndrome Patients

**DOI:** 10.3390/metabo12020124

**Published:** 2022-01-27

**Authors:** Vít Kosek, Martin Hajšl, Kamila Bechyňská, Ondřej Kučerka, Jiří Suttnar, Alžběta Hlaváčková, Jana Hajšlová, Martin Malý

**Affiliations:** 1 Department of Food Chemistry and Analysis, University of Chemistry and Technology, Technická 3, 16628 Prague, Czech Republic; kosekv@vscht.cz (V.K.); kamila.bechynska@vscht.cz (K.B.); jana.hajslova@vscht.cz (J.H.); 2 Department of Medicine, First Faculty of Medicine, Charles University in Prague and Military University Hospital, U Vojenské Nemocnice 1200, 16900 Prague, Czech Republic; martin.hajsl@uvn.cz (M.H.); ondrej.kucerka@uvn.cz (O.K.); 3 Institute of Haematology and Blood Transfusion, 12000 Prague, Czech Republic; jiri.suttnar@uhkt.cz (J.S.); alzbeta.hlavackova@uhkt.cz (A.H.)

**Keywords:** acute coronary syndrome, stroke, lipidomics, high resolution mass spectrometry

## Abstract

Lipids modified by oxidative stress are key players in atherosclerosis progression. Superimposed thrombosis with subsequent closure of the coronary artery leads to the clinical manifestation of acute coronary syndrome (ACS). While several studies focusing on alterations in lipid metabolism in the acute phase have been conducted, no information is available on patients’ lipidome alterations over longer time periods. In the current follow-up study, we analyzed plasma samples obtained from 17 patients three years after their ACS event (group AC). Originally, these patients were sampled 3–5 days after an index event (group B). Lipidome stability over time was studied by untargeted lipidomics using high performance liquid chromatography coupled to high resolution mass spectrometry (UHPLC–HRMS). Multi-dimensional statistics used for data processing indicated that plasmalogen lipids were the most prominent lipids separating the above patient groups and that they increased in the follow-up AC group. A similar trend was observed for lysophosphatidylethanolamine (LPE) and phosphatidylethanolamine (PE). The opposite trend was observed for two fatty acyls of hydroxy fatty acid (FAHFAs) lipids and free stearic acid. In addition, a decrease in the “classic” oxitadive stress marker, malondialdehyde (MDA), occurred during the follow-up period. Our findings present unique information about long-term lipidome changes in patients after ACS.

## 1. Introduction

Lipids play a crucial role in the progression of atherosclerosis. Clear evidence exists that endothelial cell activation, smooth muscle cell migration, monocyte chemotaxis, and modification of low-density lipoproteins (LDL) take part in the process triggering plaque formation in atherosclerotic lesions [1]. Oxidation of lipids and apoproteins present in LDL results in a change of lipoprotein conformation that enables penetration by the vessel wall through the endothelium modified by chronic inflammation, a process that leads to the formation of atherosclerotic plaques [2]. The plaques tend to grow, and after subsequent rupturing, blood comes into contact with the subendothelial matrix resulting in the adhesion and aggregation of platelets leading to thrombus formation [3]. Clinical manifestations of this thrombus formation are acute atherothrombotic vessel syndromes, namely acute coronary syndrome (ACS), acute ischemic stroke (AIS), and acute limb ischemia.

A number of studies concerned with the relationship between oxidative stress and the progression of acute atherothrombotic syndrome mechanisms have focused on a detailed investigation of lipidome changes and identification of markers of respective pathogenic processes. In our previous study, we analyzed plasma lipidomes in acute myocardial infarction and acute stroke patients; correlations with the levels of malondialdehyde (MDA), a routinely monitored marker of oxidative stress, were searched. For a comprehensive lipidome analysis, liquid chromatography coupled to high-resolution accurate mass spectrometry (LC-HRMS/MS) was used. The resulting data showed that lysophosphoinositol (LPI) levels were significantly higher in ACS patients’ plasma, indicating platelet activation. Higher levels of fatty acyl esters of hydroxy fatty acids (FAHFA) in controls might be due to higher anti-inflammatory activity [4].

In evaluating the significant lipidome changes associated with ACS, unanswered question regarding their duration did emerge. To learn more about lipidome “stability”, (meaning whether it is affected by ACS even several years after the event or whether only short-term effects took place and then the lipids profile returned to normal), plasma samples from respective patients were obtained for further follow-up investigations.

## 2. Results

### 2.1. Multivariate Analysis for Data Overview

**Figure 1:**
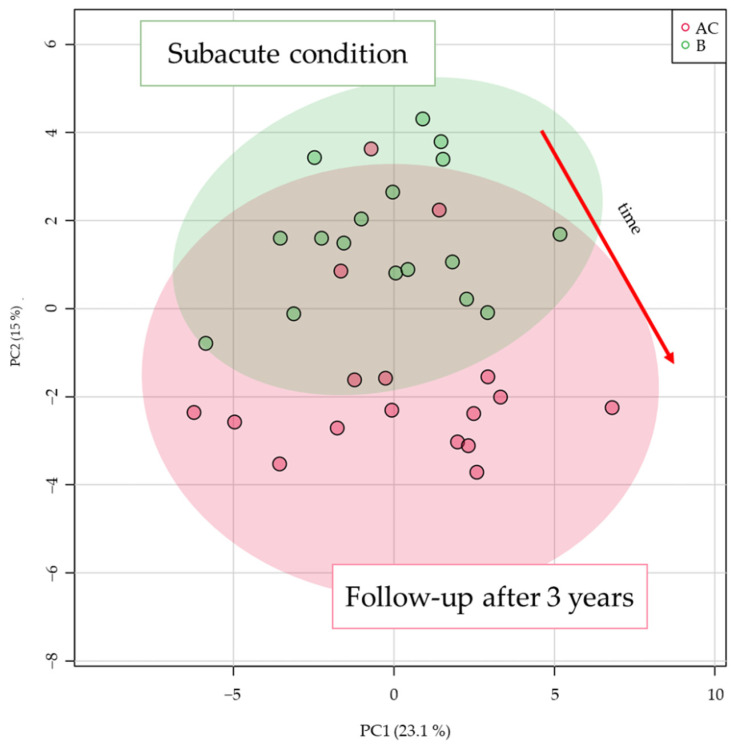
Principal component analysis (PCA) score plot based on all lipid species detected by liquid chromatography coupled to high-resolution accurate mass spectrometry (LC-HRMS) analysis.

The principal component analysis (PCA) score plots were first checked for variance in quality control (QC) samples as they were closely grouped, and good system stability was demonstrated. Altogether, a total of 261 lipid molecules were detected in plasma samples, and tendency in their profile indicated movement from subacute to follow-up conditions as can be seen in Figure 1.

### 2.2. Time Effect for ACS Patient Lipidome

To inspect the time-dependent changes under individual conditions, the lipid data were filtered using a *t*-test with *p*-value < 0.05 (corrected with the Metaboanalyst false discovery rate method). In total, 33 lipids passed this criterion and were used to build a partial least squares discriminant analysis (PLS-DA) model. The values R2Y = 0.561 and Q2Y = 0.510 characterizing the model were not high; however, the model was validated with a permutation test (999 permutations) and cross-validated analysis of variance (CV-ANOVA) with a *p*-value of 1.56803 × 10^−3^ indicating a valid model. Lipids with a variable importance on projection (VIP) score of >1 were considered as good markers and are summarized in Table 1.

**Figure 2:**
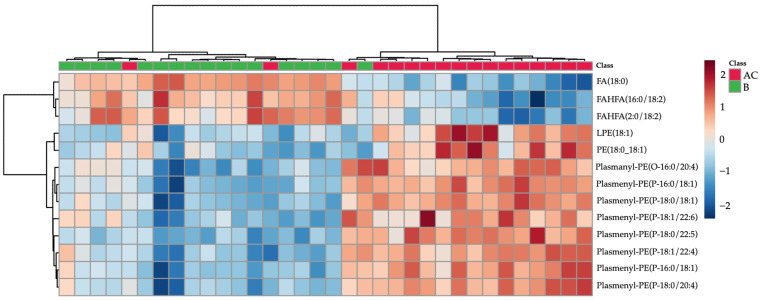
Hierarchal cluster analysis (HCA) heatmap based on lipids with *t*-test *p*-value < 0.05 and partial least squares discriminant analysis variable importance on projection (PLS-DA VIP) score > 1 (lipids characteristics shown in Table 1). Subacute patient samples are colored green, follow-up samples in red.

Plasmalogens were the most prominent lipids separating the AC and B groups and increased in the follow-up group. A similar tendency, an increase, was also observed for LPE 18:1 and PE 18:0_18:1. The opposite trend was observed for two FAHFA lipids and free stearic acid. These trends can be visualised as a heatmap in Figure 2.

### 2.3. MDA Analysis

**Figure 3:**
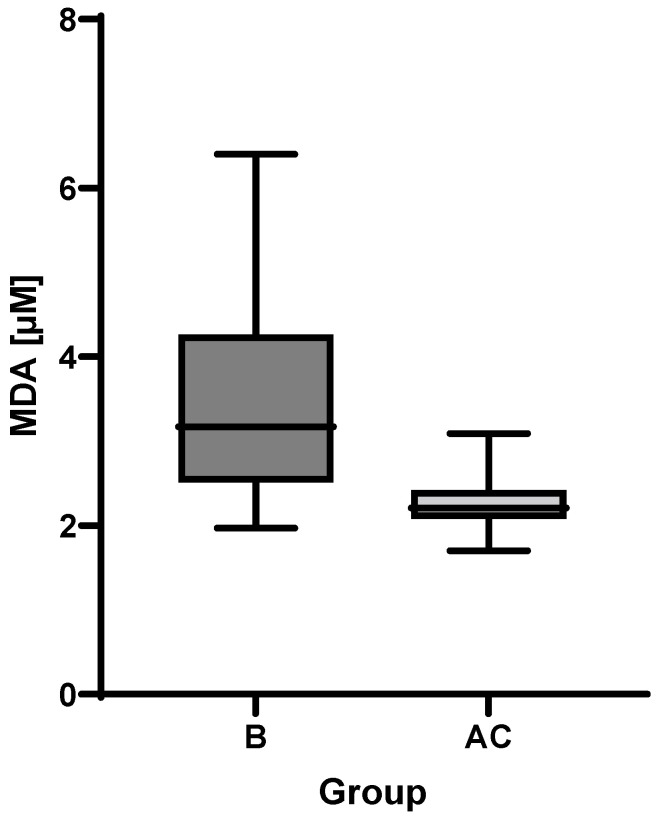
Boxplot of malondialdehyde (MDA) plasma levels in subacute (B) and follow-up (AC) groups.

MDA was analyzed in plasma samples obtained both from groups B and AC patients. In line with the expectation, the mean concentration value in the follow-up group was lower than that of subacute group as can be seen in Figure 3. Higher MDA variance in the subacute group was observed compared to the follow-up group. The Wilcoxon matched-pairs signed rank test showed a *p*-value of 0.0002.

## 3. Discussion

Results from both the lipidomics and MDA analyses suggest a return of antioxidative capacity and lowering of oxidative stress three years after the ACS event. Lower concentrations of FAHFAs also suggest lower inflammation in follow-up patients. The role of stearic acid, LPE (18:1), and PE (18:0_18:1) still requires further investigation; nevertheless, it seems that equilibrium of these lipid species is dysregulated as both LPE (18:1) and PE (18:0_18:1) levels increased in the plasma of follow-up patients.

In our previous study [4], multiple significant changes in lipid patterns were observed five days after ACS. With regards to limited data in the literature, we were interested in long-term trends in respective patients’ plasma lipidome. Several studies focusing on lipidomic characterization of patients with coronary artery disease have been conducted recently, but none of them address these long-term trends in plasma lipidome in a satisfactory manner. For example, Mundra et al. [5], who analyzed the association of plasma lipidome in patients with a history of myocardial infarction in the LIPID study, found that sphingolipids and phosphatidylserines were the most predictive class of lipids for future cardiovascular events. Because the inclusion criterion was patients’ history of myocardial infarction 3 to 36 months before involvement in the study, its results correspond to the concept of our follow-up measurements of group AC, but the results at the time of the index event are missing. In another study [6], authors focused on alterations in metabolic signature and lipid metabolism in patients with angina pectoris and myocardial infarction. Only one sampling was performed at 1.97 ± 2.67 days after the index event with no follow-up. Lysophosphatidylcholine and lysophosphatidylethanolamine (lysoPC and lysoPE, respectively) species containing unsaturated fatty acids and free fatty acids were considered as indicators of an increased risk of CAD, whereas species of lysoPC and lyso-alkyl PC containing saturated fatty acids were associated with a decreased risk. Questions arise as to whether such conclusions can be drawn without considering lipidome dynamics over time. The study by Toledo et al. [7] was concerned with the influence of a Mediterranean diet on the lipidomic profiles of patients with myocardial infarction, stroke, and/or cardiovascular death. Phosphatidylcholine 40:10, phosphatidylcholine plasmalogen 36:5a; CE (20:5), CE (20:4), and CE (22:5) and triacylglycerol 58:8 were the most prominent lipids associated with CVD risk. In this case, a 1-year follow-up sampling was performed; nevertheless, it mainly focused on the impact of dietary intervention. Under such conditions, the potential changes in lipidomes over time attributable to recovery from ACS could not be fully characterized. In an extensive study by Chen et al. [8], plasma samples obtained from 1435 patients diagnosed with coronary artery disease were analyzed. The impacted pathways included glycerophospholipid and cysteine and methionine metabolic pathways. The exact association of the index event and time of sampling was not specified; moreover, no follow-up sampling was performed. Meikle et al. [9] analyzed lipidomic profiles of plasma samples collected from patients with both stable and unstable coronary artery disease within 24 h from the index event. The most discriminating lipids were cholesterol esters, diacylglycerols, lysophosphatidylcholine, and phosphatidylinositol. Again, no other sampling was planned.

## 4. Materials and Methods

### 4.1. Study Design

Blood samples were collected from 17 patients 3–6 days after ACS (group B) and from the same patients three years after (group AC) who were diagnosed at the Central Military Hospital, University Military Hospital in Prague, Czech Republic (Table 2). These samples represent subgroups of 61 plasma samples that were analyzed from patients undergoing percutaneous coronary intervention (PCI) due to ACS described in our previous work [4]. Blood samples were collected via venipuncture. These samples were immediately centrifuged.

We analyzed samples from 17 patients originally included in the cohort of ACS patients (*n* = 17, group B). Sampling of this cohort was performed 3–5 days following ACS. The reason for this delay was to exclude the effect of heparin administered at the time of the index event due to the effects of lipoprotein lipase. The follow-up analysis was performed three years after the index event (mean 1119 ± 94 days). We planned the follow-up of 25 patients sampled in the original cohort, but one patient died, one patient refused to participate in the follow-up, and six patients could not be reached. Because of these issues, the final analysis was performed on 17 samples (*n* = 17, group AC).

Features were extracted by LipidMatch suite, which relies on MZmine 2 software for extraction, and the lipid identification was based on an in-silico fragmentation library search. At least class specific fragments were required for lipid identification. For the statistical evaluation of hundreds of features, both univariate and multivariate analysis by means of a *t*-test, hierarchical cluster analysis (HCA), and principal components analysis (PCA) was performed in both MetaboAnalyst and SIMCA.

### 4.2. Chemicals and Materials

Methanol, acetonitrile, tert-butyl methyl ether, and 2-propanol were supplied by Merck (Darmstadt, Germany). Ammonium acetate, ammonium formate, formic acid, and acetic acid were obtained from Sigma-Aldrich (Prague, Czech Republic). Click Fit 2 mL Eppendorf tubes were purchased from TreffLab (Degersheim, Germany), and 2 mL cryovials and the autosampler vials were purchased from Labicom (Olomouc, Czech Republic).

### 4.3. Sample Collection

All tested individuals agreed to participate in the study by providing informed consent. All samples were obtained and analyzed in accordance with the Ethical Committee regulations of the Military University Hospital, Prague, Czech Republic (108/11-49/2017). The study was carried out in accordance with the International Ethical Guidelines and the Declaration of Helsinki. Blood samples were drawn from the patients from all three groups in vacutainer tubes containing ethylenediaminetetraacetic acid (EDTA) and centrifuged immediately at 4000× *g* for 5 min at 4 °C. The resulting plasma samples were aliquoted into 8 tubes and stored in the dark at −80 °C until the analysis.

### 4.4. Sample Preparation

The samples were prepared by mixing 50 µL of plasma with 150 µL MeOH. After vortexing, 500 µL methyl tert-butyl ether (MTBE) was added, and the mixture was shaken for 15 min. Subsequently, 150 μL of deionized water was added to form a two-phase system. After vortexing for 30 s, the mixture was deproteinized by centrifugation at 10,000 rpm (10,621× *g*) for 10 min at room temperature. The resultant supernatants were lyophilized and stored in an −80 °C freezer if needed for later use. The freeze-dried lipid residues were resuspended in 2-propanol/methanol/deionized water (65:30:5, *v*/*v*/*v*) and used for subsequent analysis.

### 4.5. Instrumental Conditions

For the lipidomic analysis, a U-HPLC (Infinity 1290, Agilent, Santa Clara, CA, USA) coupled to a high-resolution mass spectrometer with a hyphenated quadrupole time-of-flight mass analyzer (6560 Ion Mobility Q-TOF LC/MS; Agilent) with the Agilent Jet Stream (AJS) electrospray (ESI) source was used.

For lipidomic fingerprinting, an Acquity BEH C18 (1.7 μm, 2.1 mm × 150 mm (Waters, Milford, MA, USA) was used for chromatographic separation. The chromatographic system used with ESI+ detection consisted of two mobile phases: (1) A—10 mM ammonium formate and 0.1% formic acid in acetonitrile:water (60:40, *v*/*v*) and (2) B—10 mM ammonium formate and 0.1% formic acid in 2-propanol:acetonitrile (90:10, *v*/*v*). For chromatographic separation of plasma detected in ESI- mode, two mobile phases were used: (1) A—10 mM ammonium acetate and 0.1% acetic acid in acetonitrile:water (60:40) and (2) B—10 mM ammonium acetate and 0.1% acetic acid in 2-propanol:acetonitrile (90:10, *v*/*v*). The flow rate was constant at 0.300 mL min^−1^. The column temperature was maintained at 60 °C, and the injection volume was 1 μL. The autosampler was maintained at 10 °C. Before injection, the sample injection order was randomized in MS Excel. The quality control (QC) sample was injected every 10 samples.

The mass analyzer was operated in ESI+ mode under specific conditions: (1) Gas temperature 180 °C, (2) Drying Gas 12 L/min, (3) Nebulizer pressure 40 psig, (4) Sheath gas temperature 350 °C, (5) Sheath gas flow 11 L/min, (6) capillary voltage 3000 V, (7) Nozzle voltage 250 V, (8) fragmentor voltage 380 V, and (9) octopole radiofrequency voltage 750 V. Data were acquired over the *m*/*z* range of 50 to 1700 at the rate of 2 spectra/s. The *m*/*z* range was autocorrected on reference masses 121.0509 and 922.0098.

The mass analyzer was operated in ESI mode under specific conditions: (1) Gas temperature 180 °C, (2) Drying Gas 12 L/min, (3) Nebulizer pressure 45 psig, (4) Sheath gas temperature 350 °C, (5) Sheath gas flow 11 L/min, (6) capillary voltage 3500 V, (7) Nozzle voltage 250 V, (8) fragmentor voltage 350 V, and (9) octopole radiofrequency voltage 250 V. Data were acquired over the *m*/*z* range of 50 tp 1700 at the rate of 2 spectra/s. The *m*/*z* range was autocorrected on reference masses 119.0363 and 980.0164.

The chromatographic gradient consisted of several phases: (1) For ESI+, the initial composition of 60% A and 40% B was maintained from 0 to 2 min, (2) from 2 to 4 min, the initial composition was ramped to 50% A and 50% B, and (3) from 4 to 5 min to 60% B, and (4) at 15 min, the composition was increased to 100% B. These steps were followed by flushing for 3 min with the initial solution to re-equilibrate the column. For ESI analysis, the initial composition of 60% A and 40% B was maintained from 0 to 2 min, from 2 to 4 min, the initial composition was ramped to 50% A and 50% B, from 4 to 5 min to 60% B, and at 12 min, the composition was increased to 80% B after which the composition was immediately set to 100% B until 15 min to remove triacylglycerols from the column. These steps were followed by 3 min of flushing the column with the initial phase to re-equilibrate the column.

MDA analysis was based on an already published method [4].

### 4.6. Data Processing for Fingerprinting Experiment

The data were processed using the LipidMatch [8] suite, which uses MZmine 2 for feature extraction and an R script for lipid identification. A custom-built R script based on a MetaboAnalystR package was used to filter out features based on their univariate statistics. Statistically insignificant compounds were filtered out if they did not meet the criterion of an ANOVA *p*-value of <0.05. These data were then loaded by SIMCA, where statistical models were built. When building PLS-DA and OPLS-DA models in SIMCA, logarithmic transformation and pareto scaling were used to ensure a higher significance of low abundant compounds. Lipids were identified based on fragmentation spectra and accurate mass in silico libraries which are a part of LipidMatch suite. Fragmentation spectra of the significant compounds were also compared to those present in METLIN and LIPIDMAPS databases and their identities confirmed. Univariate statistics with MDA were performed in GraphPad Prism version 9.3.0.

## 5. Limitations of the Study

The main limitation of the study is a small number of patients. A bigger cohort would be better from a statistical point of view; nevertheless, the current study should be understood as a proof of concept. Moreover, under common conditions of clinical practice (and specifically in a pandemic), it is extremely difficult to recall a sufficient number of patients willing to cooperate after three years. The strength of this study is the long-term follow-up. From the point of view of searching specific markers, several studies concerned with a search for ACS prognostic markers reported compounds other than lipids, for instance troponin, a protein which is released from cardiomyocytes during necrosis. Regarding lipids, the well-known LDL cholesterol has a prognostic impact. However, the other lipids identified within lipidomic research (mentioned in the manuscript) have never been confirmed as markers in follow-up clinical studies. Contrary to that, the presented study provides more realistic information on conceivable lipidic markers.

## 6. Conclusions

Although the cohort of patients re-called three years after ACS was rather limited, the data obtained by UHPLC HRMS-based plasma lipidomics provided some interesting results:

- The concentration of MDA, an oxidative stress marker, decreased, and plasma antioxidant capacity, as indicated by higher levels of plasmalogens, increased.
- The observed increase in FAHFA class lipids in samples of subacute patients may be indicative of the inflammation that accompanies ACS; a subsequent decrease of these lipids in follow-up samples was observed.
- Further investigation of lipidome changes in a larger cohort over a longer period of time might confirm the above observations, and possibly complement existing knowledge.
- The lipidome changes attributable strictly to ACS seems to be very limited in long-term basis.

## Figures and Tables

**Table 1:** List of detected lipids with variable importance on projection (VIP) score > 1 from a partial least squares discriminant analysis (PLS-DA) model built on lipids which passed a *t*-test *p*-value (FDR corrected) < 0.05 criterion and their characteristics.

Lipid Name	Detected Adduct	Retention Time [min]	*m*/*z* Value	*t*-Test FDR *p*-Value	PLS-DA VIP Score
Plasmenyl-PE(P-18:0/22:5)	[M − H]^−^	10.48	776.5602	3.94 × 10^−5^	1.31
Plasmenyl-PE(P-18:0/20:4)	[M − H]^−^	10.52	750.5422	7.51 × 10^−5^	1.28
Plasmenyl-PE(P-18:0/18:1)	[M − H]^−^	11.43	728.5593	1.54 × 10^−4^	1.24
Plasmenyl-PE(P-16:0/20:4)	[M − H]^−^	9.643	722.5105	7.51 × 10^−5^	1.24
Plasmenyl-PE(P-16:0/18:1)	[M − H]^−^	10.59	700.5269	1.54 × 10^−4^	1.23
Plasmenyl-PE(P-18:1/20:4)	[M − H]^−^	9.658	748.5265	8.33 × 10^−4^	1.20
LPE(18:1)	[M − H]^−^	3.013	478.2923	2.83 × 10^−4^	1.09
Plasmenyl-PE(O-16:0/20:4)	[M − H]^−^	9.982	724.5262	4.11 × 10^−3^	1.08
FAHFA(16:0/18:2)	[M − H]^−^	5.078	533.4528	5.55 × 10^−3^	1.07
Plasmenyl-PE(P-18:1/22:6)	[M − H]^−^	9.359	772.5261	2.52 × 10^−3^	1.05
PE(18:0_18:1)	[M − H]^−^	10.99	744.5526	1.62 × 10^−4^	1.03
FAHFA(2:0/18:2)	[M − H]^−^	5.078	337.2339	2.23 × 10^−2^	1.02
FA(18:0)	[M − H]^−^	6.535	283.2636	4.94 × 10^−2^	1.01

**Table 2:** Characteristics of the study group.

Patient Characteristics	Group B (*n* = 17)
Age (y)	61
Sex (m/f)	12/5
Clinical characterization	
Arterial hypertension (*n*/%)	11/65
Diabetes mellitus (*n*/%)	4/24
Current smoker (*n*/%)	9/53
BMI	29.2
Medical history	
History of MI (*n*/%)	1/6
History of PCI (*n*/%)	1/6
Laboratory results	
CKD epi (mL/min)	88.7
Kreatinin (µmol/L)	75
Total cholesterol (mmol/L)	4.5
TAG (mmol/L)	1.1
LDL cholesterol (mmol/L)	2.9
HDL cholesterol (mmol/L)	1.1
Post-procedure hypolidimic treatment	
Statin (*n*/%)	17/100

BMI—body mass index; MI—myocardial infarction; PCI—percutaneous coronary intervention; CABG—coronary artery bypass graft; CKD epi—glomerular filtration; TAG—triacylglycerols; LDL—low density lipoproteins; HDL—high density lipoproteins.

## Data Availability

Data available on request due to restrictions eg privacy or ethical. The data presented in this study are available on request from the corresponding author. The data are not publicly available due to ethical reasons.
